# Supplementary material for: A Global Screen for Assembly State Changes of the Mitotic Proteome by SEC-SWATH-MS
Source: Cell Syst. 2020 Feb 26;10(2):133–155.e6. doi: 10.1016/j.cels.2020.01.001 (PMC7042714; doi:10.1016/j.cels.2020.01.001)

Q9Y4C1 | KDM3A_HUMAN | KDM3A JHDM2A JMJD1 JMJD1A KIAA0742 TSGA
Monomer MW [kDa]: 147.341 Monomer expected elution fraction: 37

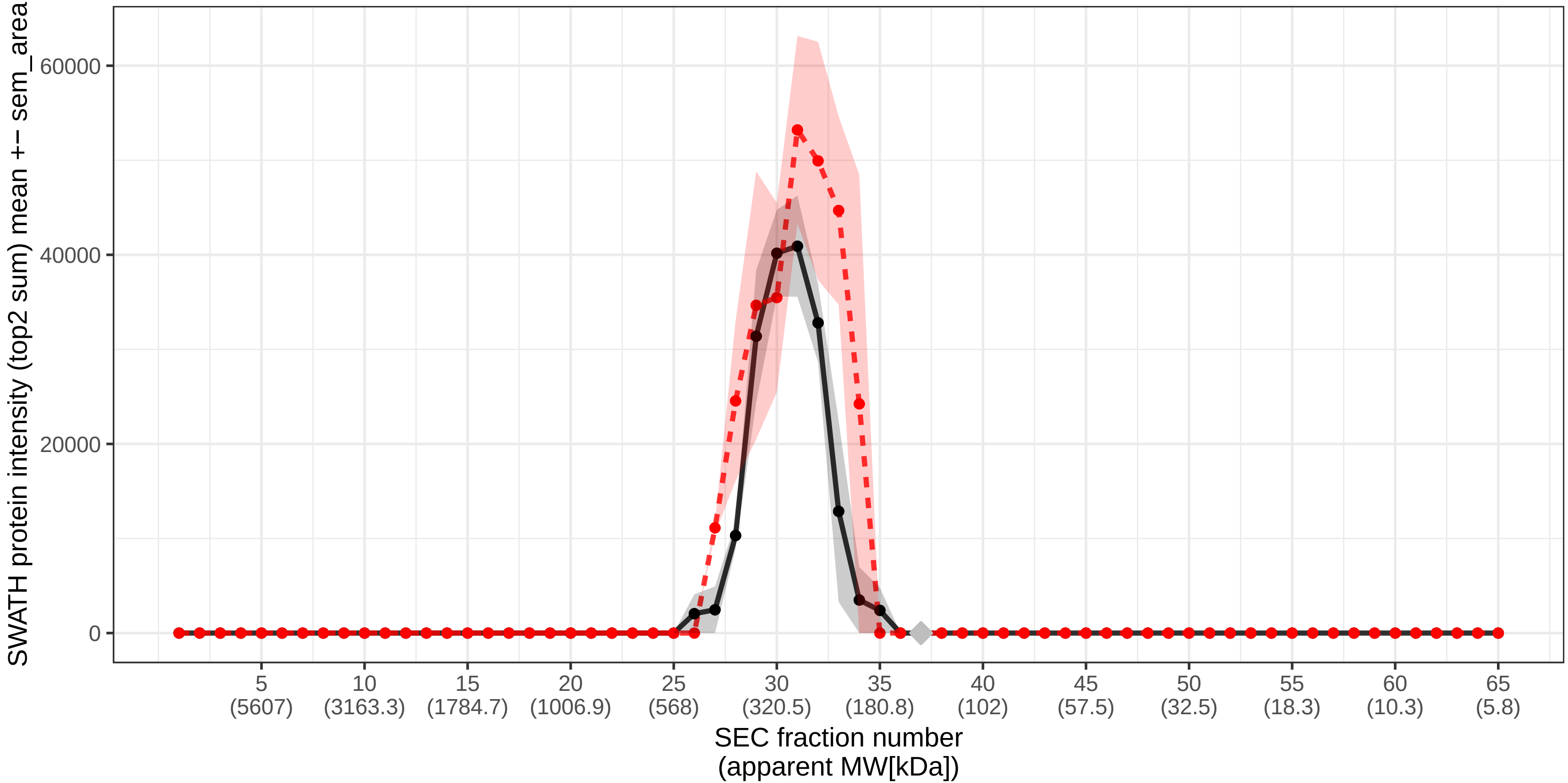

Supplement: Data S1. SEC-SWATH-MS Protein Chromatograms, Related to Figure 1 [file mmc6.zip › SECchrom_Q9Y4C1_KDM3A_HUMAN_KDM3A_JHDM2A_JMJD1_JMJD1A_KIAA0.pdf]
